# HIV-1 Vpu and HIV-2 Env counteract BST-2/tetherin by sequestration in a perinuclear compartment

**DOI:** 10.1186/1742-4690-7-51

**Published:** 2010-06-07

**Authors:** Heiko Hauser, Lisa A Lopez, Su Jung Yang, Jill E Oldenburg, Colin M Exline, John C Guatelli, Paula M Cannon

**Affiliations:** 1 Department of Molecular Microbiology and Immunology, Keck School of Medicine of the University of Southern California, Los Angeles, California, USA; 2 Department of Medicine, University of California San Diego, La Jolla, California, USA; 3 San Diego Veterans Affairs Healthcare System, San Diego, California, USA

## Abstract

**Background:**

In the absence of the Vpu protein, newly formed HIV-1 particles can remain attached to the surface of human cells due to the action of an interferon-inducible cellular restriction factor, BST-2/tetherin. Tetherin also restricts the release of other enveloped viral particles and is counteracted by a several viral anti-tetherin factors including the HIV-2 Env, SIV Nef and KSHV K5 proteins.

**Results:**

We observed that a fraction of tetherin is located at the surface of restricting cells, and that co-expression of both HIV-1 Vpu and HIV-2 Env reduced this population. In addition, Vpu, but not the HIV-2 Env, reduced total cellular levels of tetherin. An additional effect observed for both Vpu and the HIV-2 Env was to redirect tetherin to an intracellular perinuclear compartment that overlapped with markers for the TGN (*trans*-Golgi network). Sequestration of tetherin in this compartment was independent of tetherin's normal endocytosis trafficking pathway.

**Conclusions:**

Both HIV-1 Vpu and HIV-2 Env redirect tetherin away from the cell surface and sequester the protein in a perinuclear compartment, which likely blocks the action of this cellular restriction factor. Vpu also promotes the degradation of tetherin, suggesting that it uses more than one mechanism to counteract tetherin restriction.

## Introduction

Viral pathogens frequently disable components of both intrinsic and adaptive host immune responses. The human immunodeficiency virus (HIV) expresses accessory proteins that play essential roles to counteract such host defenses [1]. Strategies include targeting the host anti-viral proteins or restriction factors for degradation through the recruitment of cullin-RING finger ubiquitin ligases, as occurs when Vif counteracts APOBEC3G, or Vpu targets CD4. Alternatively, the trafficking pathways used by the host factors can be altered to prevent expression at the cell surface, as occurs with Nef and CD4 or MHC class I. The HIV-1 Vpu protein also counteracts an α-interferon-inducible host cell restriction, BST-2/CD317/HM1.24 ("tetherin"), that prevents the release of newly formed virions from the cell surface [2-4]. Virions lacking Vpu accumulate at the cell surface and in intracellular compartments, leading to a correspondingly reduced ability of the virus to spread [3,5,6].

Tetherin restriction of virus release is also active against other enveloped viruses including retroviruses, filoviruses and arenaviruses, suggesting that it constitutes a broadly-acting host defense mechanism [7-10]. It is therefore likely that successful pathogens will have evolved effective counteracting strategies, and several different proteins from RNA viruses have now been shown to counteract tetherin restriction, including the HIV-1 Vpu, HIV-2 Env, and Ebola GP proteins that target human tetherin [3,4,7,11-13], and the SIV Nef protein that is active against the form of the protein in Old World primates [14-17]. Tetherin is also targeted for degradation by the K5 protein from Kaposi's sarcoma associated herpesvirus (KSHV), an E3 ubiquitin ligase that reduces both total and cell surface levels of the protein [18,19]. Since K5 activity is necessary for efficient KSHV release [19], this suggests that tetherin restriction is also active against enveloped DNA viruses.

Tetherin is an unusual membrane protein, containing both an N-terminal transmembrane domain and a C-terminal GPI anchor, and it is able to form cysteine-linked homodimers [20,21]. It has been suggested that tetherin could retain viruses at the cell surface by physically linking the viral and plasma membranes [3,22]. Consequently, removal of tetherin from the cell surface could be the basis of Vpu's antagonism [4], although such a model has been challenged [23]. Steady-state levels of tetherin are reduced in the presence of Vpu [15,24,25]. It has been suggested that this occurs by recruitment of an SCF-E3 ubiquitin ligase complex, through an interaction between the β-TrCP protein and conserved phospho-serine residues in Vpu's cytoplasmic tail. Ubiquitinylation of tetherin could then lead to either proteasomal degradation [24], or internalization into endo-lysosomal pathways [25-27].

In the current study, we analyzed the ability of the HIV-1 Vpu and HIV-2 Env to overcome tetherin restriction. In agreement with previous reports, we found that both proteins removed tetherin from the cell surface, and that additionally Vpu, but not HIV-2 Env, reduced total cellular levels of tetherin. Interestingly, both proteins also concentrated tetherin in a perinuclear compartment that overlapped with markers of the *trans*-Golgi network (TGN). We hypothesize that in addition to targeting tetherin for degradation, Vpu may use a mechanism in common with HIV-2 Env to sequester tetherin away from site of virus assembly and thereby counteract its activity.

## Results

### Tetherin is present at the cell surface and in a perinuclear compartment

**Figure 1:**
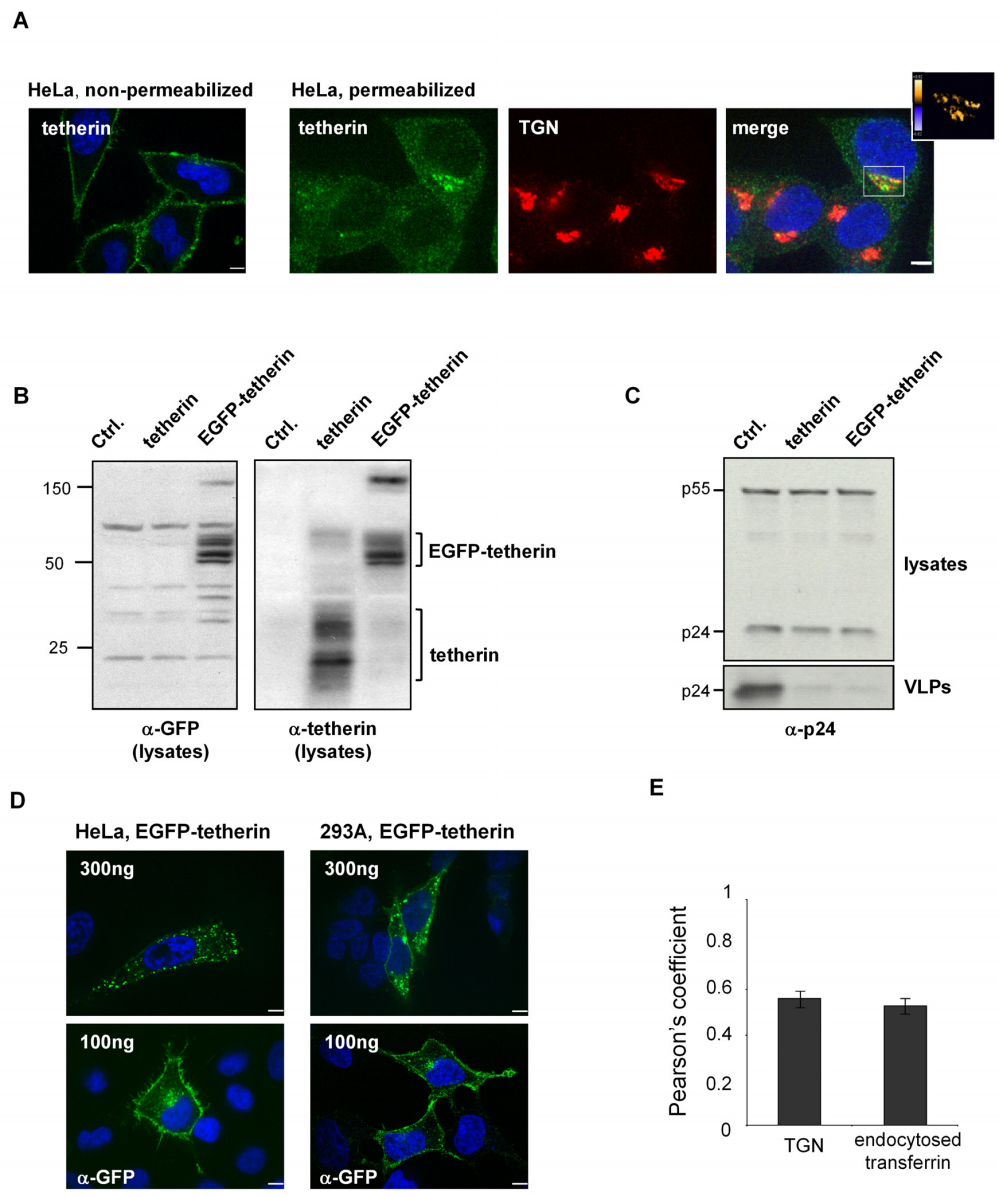
**Cellular distribution of tetherin**. **(A) **Confocal analysis of HeLa cells showing the distribution of endogenous tetherin, detected with a specific antiserum. Cells that were fixed but not permeablized (left panel) allowed visualization of tetherin at the cell surface, while permeabilized cells revealed tetherin concentrated in a perinuclear compartment that was visible in ~50% of cells. This intracellular pool co-localized with a marker for the TGN (TGN-46), as shown by the PDM analysis in the upper right corner of the merged image, where positive co-localization is pseudocolored in orange. Scale bars represent 10 μM. **(B) **293A cells were co-transfected with 10 μg HIV-1-pack and 100 ng of expression plasmids for either untagged tetherin or EGFP-tagged tetherin. Cell lysates were analyzed by Western blotting, using antibodies against GFP and tetherin. **(C) **Cell lysates and pelleted supernatant fractions (VLPs) from same experiment as **(B) **were probed for HIV-1 p24 expression. Both tetherin constructs inhibited VLP release. **(D) **HeLa and 293A cells were transfected with either 100 ng or 300 ng of the EGFP-tetherin plasmid. With 300 ng, a punctate pattern of EGFP fluorescence was observed throughout the cells; with 100 ng, the protein could only be detected using an anti-GFP antibody, that revealed an intense surface rim and a fainter PNC in both types of cells. Cells were fixed and permeabilized before staining. Scale bars represent 10 μM. **(E) **The intracellular concentration of EGFP-tetherin in transiently transfected HeLa cells (100 ng plasmid) was analyzed by confocal microscopy using anti-GFP antibody and specific markers for the TGN (TGN46) and recycling endosomes (endocytosed transferrin). The degree of co-localization was calculated using Pearson's coefficients. Mean +/- SEM is shown for 20 individual cells analyzed.

It has been suggested that tetherin could retain viruses at the cell surface by physically linking viral and plasma membranes [3,22]. A correlate of such a model is that at least a fraction of the protein should be present at the plasma membrane. Previous studies of rat and mouse tetherin have shown that the protein recycles between the plasma membrane and a perinuclear compartment that overlaps with cellular markers for the TGN [20,28], while human tetherin has been partially co-localized with both the TGN and recycling endosomes [29,30]. We analyzed the distribution of tetherin in HeLa cells by confocal microscopy using both permeabilized cells to observe the localization of intracellular protein, and non-permeabilized cells, which allowed a clearer visualization of the cell surface population. We found tetherin at the surface of all cells analyzed (Figure 1A). In addition, about half of the cells also displayed an intracellular concentration in a perinuclear compartment that co-localized with a TGN marker.

We also examined the distribution of exogenously expressed tetherin, introduced by transient transfection of cells with either native or N-terminal EGFP-tagged versions of human tetherin (Figure 1B). EGFP-tetherin was also able to restrict the release of HIV-1 virus-like particles (VLPs) following transfection into 293A cells, which are normally non-restrictive (Figure 1C). Confocal analysis of EGFP-tetherin distribution in transfected HeLa or 293A cells, detected using EGFP autofluorescence, revealed a highly punctate pattern (Figure 1D), but these studies required us to transfect considerably more plasmid DNA (300 ng) than was necessary to achieve full restriction of VLP release (<100 ng). Therefore, in order to visualize the distribution of EGFP-tetherin at the lower levels of expression that were sufficient to profoundly restrict VLP release, we transfected 100 ng of the EGFP-tetherin plasmid and detected the protein using an anti-GFP antibody. Under these conditions, EGFP-tetherin was observed at the plasma membrane and also intracellularly, in a distribution that was similar to that observed for the endogenous protein in HeLa cells (Figure 1D). Co-labeling experiments determined that the intracellular population of tetherin overlapped extensively with markers (Figure 1E), suggesting that tetherin populates these vesicles as it traffics between the TGN and the plasma membrane.

### Removal of tetherin from cell surface by HIV anti-tetherin factors

**Figure 2:**
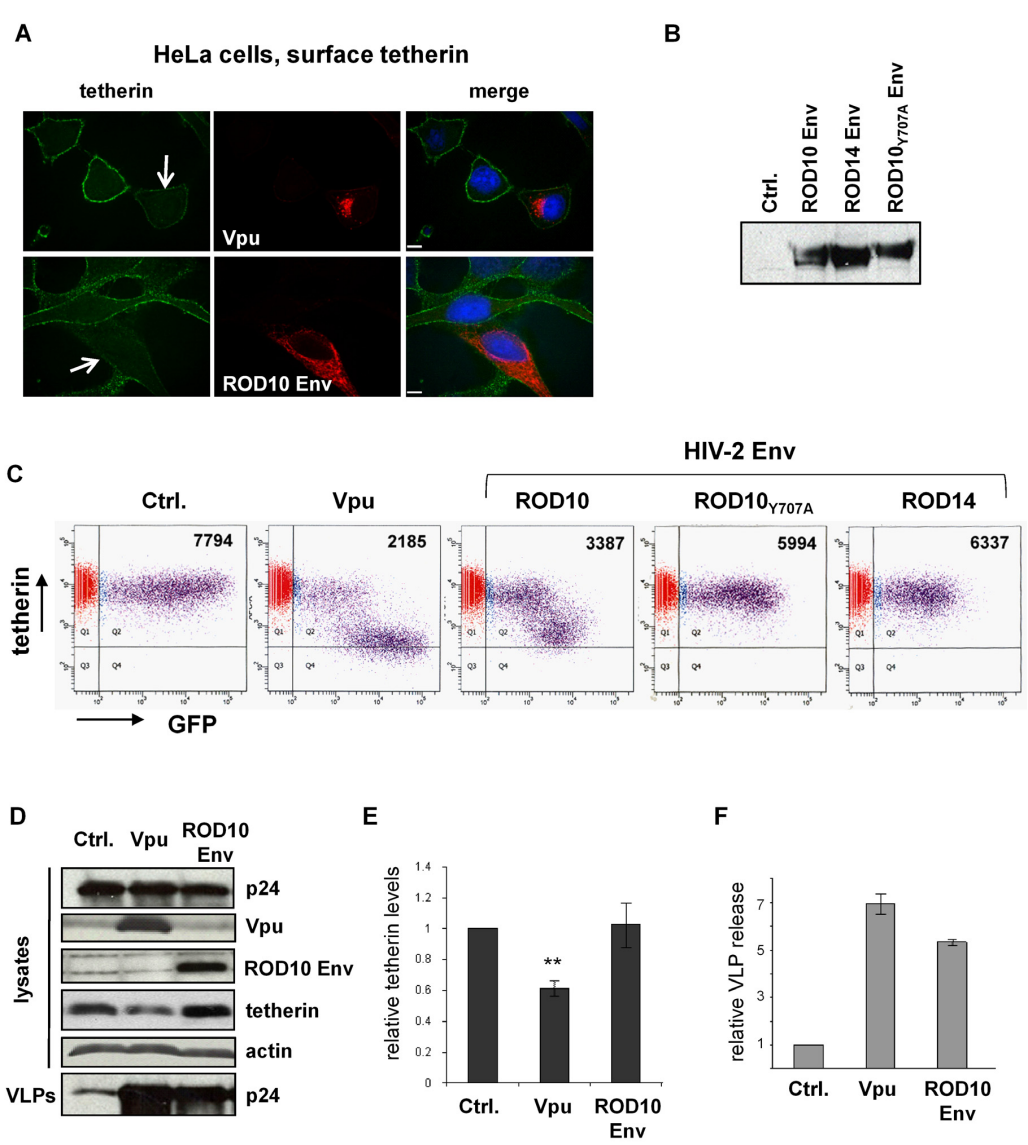
**Effect of HIV-1 Vpu and HIV-2 Env on tetherin**. **(A) **HeLa cells were transfected with 2 μg of either a Vpu expression plasmid (pcDNA-Vphu) or a ROD10 HIV-2 Env expression plasmid and analyzed by confocal microscopy. Cell surface tetherin was detected by addition of an anti-tetherin antibody prior to fixation and permeabilization, while incubation with anti-Vpu or anti-Env antibodies was performed after permeabilization. The cell surface rim of tetherin was reduced in cells co-expressing Vpu or ROD10 Env (arrowed cells). Scale bars represent 10 μM. **(B) **HeLa cells were co-transfected with 10 μg of pHIV-1-pack, together with 2 μg of expression plasmids for HIV-2 Env ROD10, ROD10_Y707A _or ROD14. Proteins in cell lysates were analyzed by Western blotting using an anti-HIV-2 Env antibody. **(C) **FACS analyses of HeLa-CD4 P4.R5 cells transfected with a plasmid expressing GFP, together with either an empty vector control (Ctrl.), Vpu (pcDNA-Vphu), or Env-expression vectors from HIV-2 ROD10, ROD10_Y707A _or ROD14. Staining for tetherin with HM1.24 monoclonal antibody and gating on the GFP-expressing population allowed for enrichment of cells that had been transfected. The mean fluorescence intensity of tetherin staining is shown for the GFP-expressing population. **(D) **HeLa cells were co-transfected with 10 μg of pHIV-1-pack, together with 2 μg of expression plasmids for Vpu (pcDNA-Vphu) or the ROD10 Env. Proteins in cell lysates or VLPs were analyzed by Western blotting as indicated. Lysates were deglycosylated prior to analysis of tetherin. **(E) **Mean relative levels of tetherin in lysates of HeLa cells expressing Vpu or ROD10 Env. Error bars represent SEM. ** indicates statistical significance, p < 0.01 compared to control, non-transfected cells, n = 9. **(F) **Mean relative level of VLP release from HeLa cells expressing Vpu or ROD10 Env, calculated as the ratio of p24 signal in VLPs:lysates, made relative to the pHIV-1-pack control (Ctrl.). Error bars represent SEM, n = 7.

The expression of Vpu or HIV-2 Env has previously been reported to reduce the amount of tetherin detected at the cell surface [4,13]. We examined the effects of HIV-1 Vpu and HIV-2 Env (from the ROD10 isolate) on the cell surface levels of endogenous tetherin present in HeLa cells, using confocal microscopy of non-permeablized cells, where we observed that both proteins were able to reduce surface tetherin (Figure 2A). These findings were corroborated by FACS analysis, where we further observed that the ROD14 and ROD10_Y707A _variants of the HIV-2 Env (Figure 2B), that we have previously shown to be defective at enhancing HIV-1 VLP release [7], did not significantly reduce cell surface tetherin (Figure 2C).

A common strategy used by viruses to neutralize host antiviral factors is to promote their degradation through proteasomal or lysosomal pathways. We therefore also compared the effects of the HIV proteins on total cellular levels of tetherin. Endogenous tetherin appeared as multiple bands on a Western blot, ranging in size between approximately 26 and 35 kDa, (data not shown), and treatment of cell lysates with PNGase to remove N-linked glycans produced a faster-running species of about 20 kDa (Figure 2D). As previously reported [13,18], we found that Vpu reduced steady state levels while the ROD10 Env had no effect (Figure 2E). Finally, we confirmed the ability of Vpu and ROD10 Env to enhance VLP release from HeLa cells using the same transfection conditions and time of analysis as were used in all other assays (Figure 2F).

### HIV anti-tetherin factors promote intracellular sequestration of tetherin

**Figure 3:**
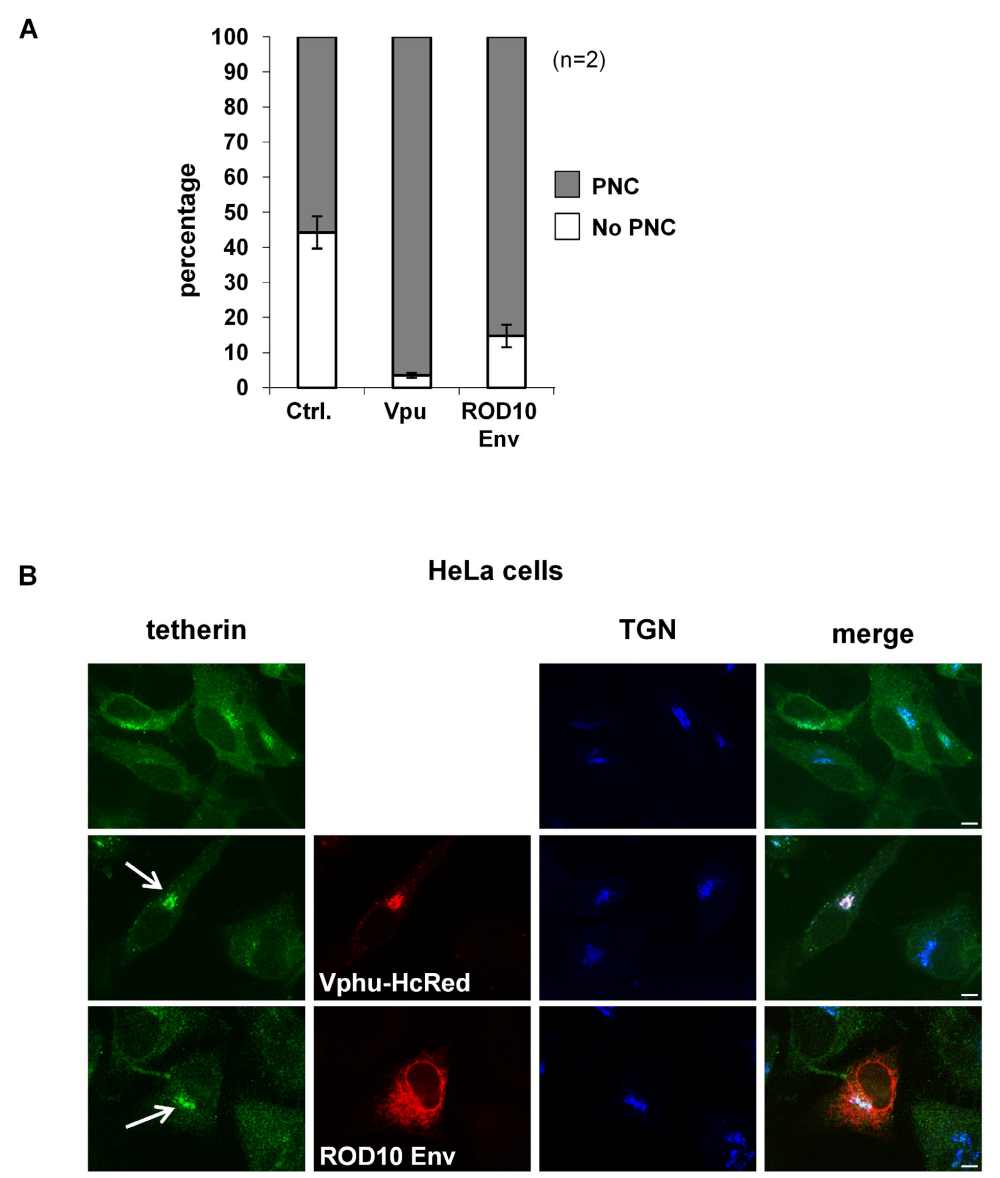
**Redistribution of tetherin to an intracellular compartment by HIV anti-tetherin factors**. **(A) **The percentage of HeLa cells displaying tetherin concentrated in a perinuclear compartment (PNC) was calculated for 100 cells, from either control (Ctrl.) cells or cells transfected with 2 μg of Vpu or ROD10 Env expression plasmids. Mean +/- SEM is shown for n = 2 independent experiments. **(B) **HeLa cells transfected with either Vpu (Vphu-HcRed) or ROD10 Env, showed increased concentration of tetherin in a perinuclear compartment (arrowed), that co-stained with the TGN marker, TGN46. The triple color merged image is shown. Scale bars represent 10 μM.

**Figure 4:**
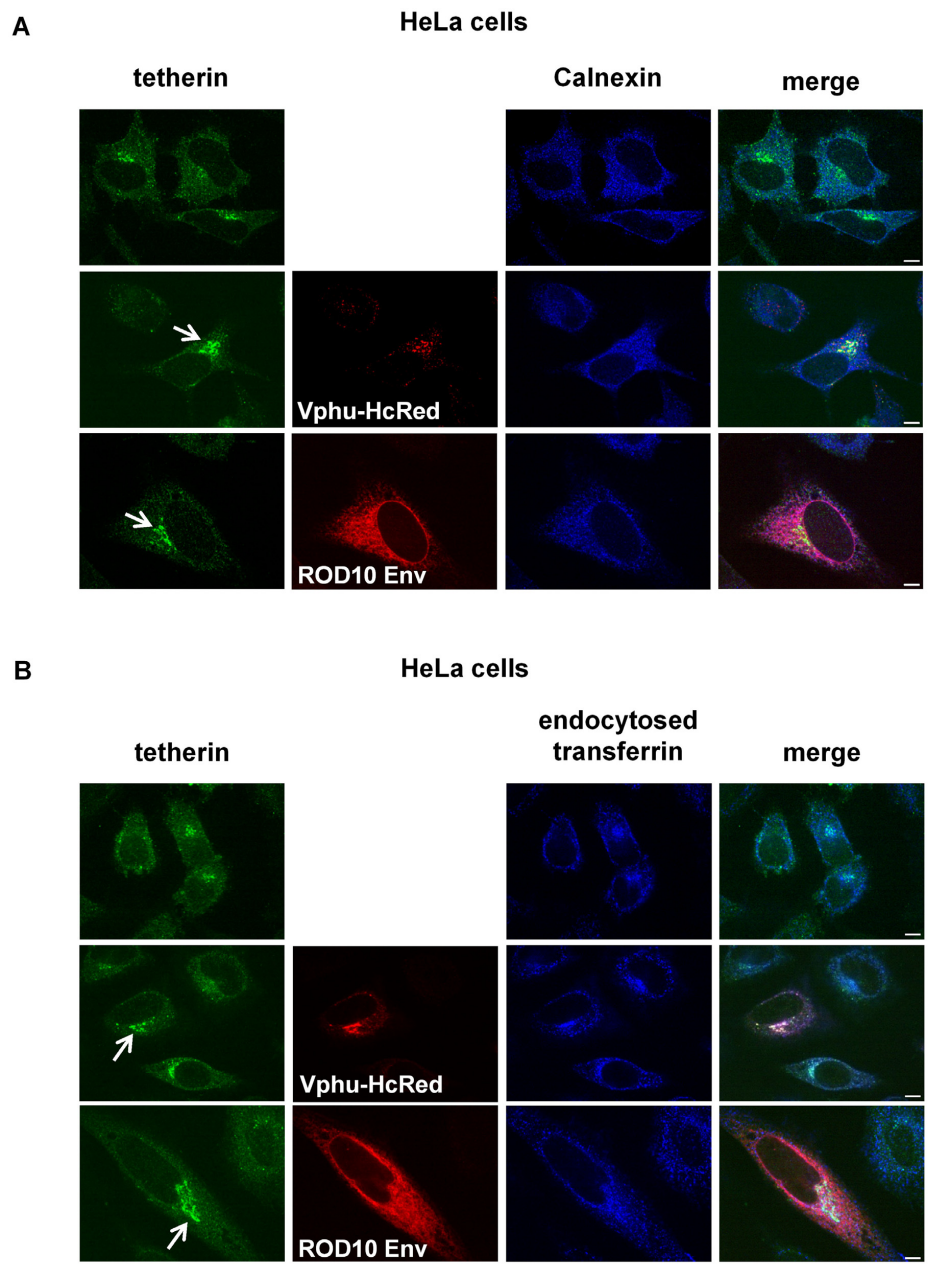
**Co-staining of tetherin with calnexin and endocytosed transferrin**. HeLa cells transfected with either 2 μg of Vpu (Vphu-HcRed) or ROD10 Env plasmids were analyzed for co-localization with the ER marker, calnexin **(A)**, or with endocytosed transferrin **(B)**. Triple color merged images are shown. Scale bars represent 10 μM.

We examined the effects of Vpu and ROD10 Env on the intracellular distribution of tetherin. Tetherin in control HeLa cells was present in a perinuclear compartment in approximately 50% of cells, but this fraction was significantly increased in the presence of both Vpu and the ROD10 Env (Figure 3A). In both cases, this intracellular tetherin co-localized strongly with a marker for the TGN (Figure 3B), but not with an ER marker (Figure 4A), and that there was partial overlap with endocytosed transferrin (Figure 4B). Vpu also co-localized strongly with tetherin in this compartment, and although a minority of the ROD10 Env population co-localized with the TGN or endocytosed transferrin markers, the majority of the Env protein was present in the ER and did not overlap with tetherin.

**Figure 5:**
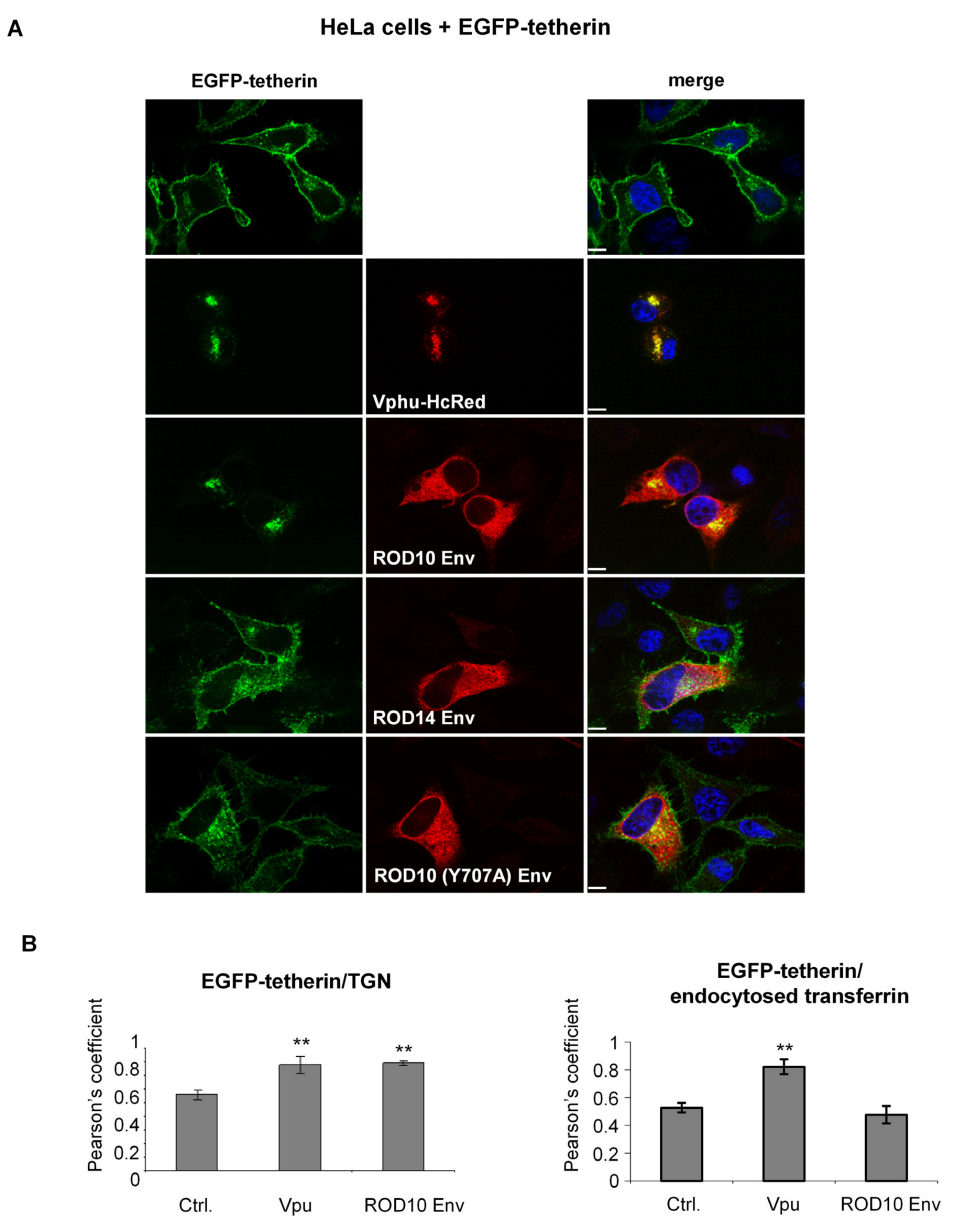
**Redistribution of EGFP-tetherin by functional anti-tetherin factors**. **(A) **HeLa cells were co-transfected with 100 ng of EGFP-tetherin and the indicated HIV proteins. EGFP-tetherin was detected using an anti-GFP antibody, and was found to be removed from the cell surface and concentrated internally by expression of both Vpu (Vphu-HcRed) and ROD10 Env. The non-functional Env proteins from ROD14 or ROD10(Y707A) had no effect on cell surface EGFP-tetherin levels, although we frequently observed that the EGFP-tetherin signal was brighter with more visible intracellular puncta in the co-transfected cells. Scale bars represent 10 μM. **(B) **The degree of co-localization of EGFP-tetherin with markers for the TGN or endocytosed transferrin, in the presence of Vpu or ROD10 Env, was calculated using Pearson's coefficients. Statistical significance was calculated using unpaired t-tests, ** indicates p < 0.01 compared to control, non-transfected cells.

The effects we observed with native tetherin were also observed using EGFP-tetherin transfected into HeLa cells, where the presence of Vpu or the ROD10 Env completely removed the cell surface protein and caused tetherin to be highly concentrated in the perinuclear compartment (Figure 5A). In contrast, the non-functional ROD14 and ROD10_Y707A _Envs did not affect the overall distribution of EGFP-tetherin, although we did note that the EGFP signal was frequently brighter in their presence, and more intracellular puncta were visible in cells co-expressing these Envs. Tetherin co-localized even more strongly with markers for the TGN in the presence of Vpu and ROD10 Env, while Vpu, but not ROD10 Env, increased tetherin's co-localization with endocytosed transferrin (Figure 5B). Finally, we confirmed that the effects seen with EGFP-tetherin were not a consequence of the N-terminal EGFP tag since untagged tetherin transfected into 293A cells, which do not express detectable endogenous tetherin, was also relocated to a perinuclear compartment by Vpu or ROD10 Env (data not shown).

### Redistribution of tetherin is a specific effect

**Figure 6:**
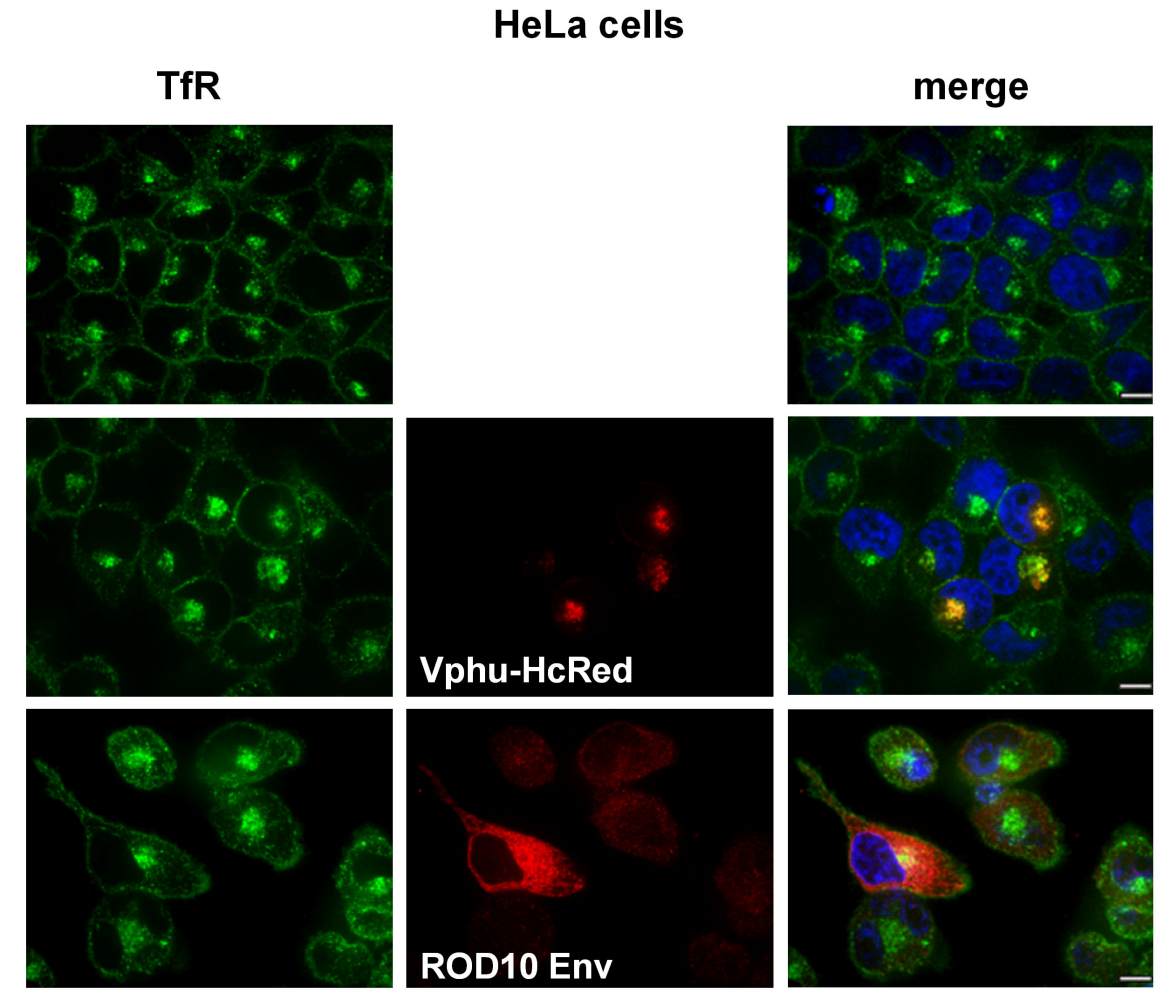
**Vpu and ROD10 Env have no effect on TfR distribution**. HeLa cells were either mock treated or transfected with 2 μg Vphu-HcRed or 2 μg ROD10 Env expression plasmids, permeabilized and stained with specific antibodies against human transferrin receptor (TfR) or HIV-2 Env, or visualized by HcRed fluorescence, and analyzed by confocal microscopy. TfR was found at the cell surface and in a perinuclear concentration, and its distribution was unaltered by expression of either viral protein. Scale bars represent 10 μm.

To determine whether the relocalization of tetherin caused by Vpu or ROD10 Env was a specific interaction between the proteins, or the result of a more global effect on protein trafficking, we analyzed the effects of expression of Vpu and ROD10 Env on the distribution of the human transferrin receptor 1 (TfR1). Like tetherin, TfR1 is a type II membrane protein, although it does not contain a GPI anchor or co-localize to lipid rafts. In control, non-transfected HeLa cells, TfR1 was present at the cell surface and in a perinuclear compartment. Co-expression of Vpu or ROD10 Env had no effect on its distribution (Figure 6), indicating that the ability of these HIV proteins to remove tetherin from the cell surface is a specific interaction.

### Tetherin redistribution by HIV-1 and HIV-2 proviral clones

**Figure 7:**
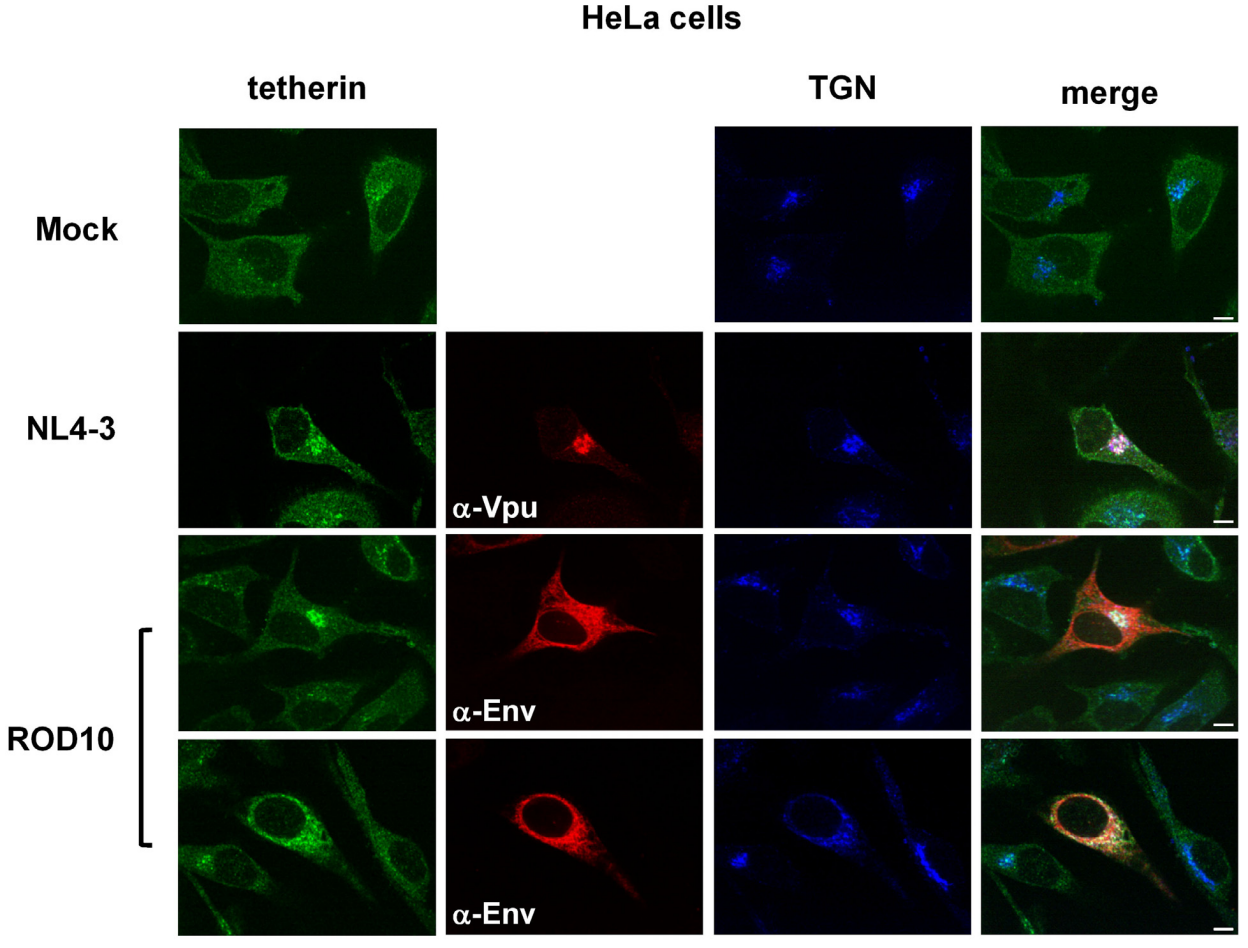
**Tetherin redistribution by HIV-1 and HIV-2 proviral clones**. HeLa cells were either mock treated or transfected with 8 μg of HIV-1_NL4-3 _or HIV-2_ROD10 _proviral clones. Cells were fixed, permeabilized, and stained for endogenous tetherin (green), the TGN46 marker (blue), HIV-1 Vpu (red) or HIV-2 Env (red). Triple color merged images are shown. NL4-3 transfected cells showed tetherin co-localized with Vpu and the TGN. ROD10 transfected cells had two distinct appearances. ~25% of cells showed tetherin localized with a compact TGN marker (upper panels), while the majority of the cells had tetherin in a more diffuse perinuclear location that co-localized with more distorted TGN staining (lower panels). Scale bars represent 10 μM.

We analyzed the distribution of tetherin in HeLa cells transfected with proviral clones of HIV-1_NL4-3 _and HIV-2_ROD10_. Similar to the situation we observed with the Vpu and HIV-2 Env expression plasmids, tetherin was found to be redistributed to an intracellular compartment that overlapped with a TGN marker (Figure 7). Interestingly, for cells transfected with the HIV-2 clone, although tetherin continued to overlap strongly with the TGN marker, the appearance of this organelle was distorted in the majority of cells, so that only ~25% of the cells had a typical TGN appearance and exhibited a compact tetherin perinuclear concentration (Figure 7, ROD10 upper panel). However, even in the cells that had a more dispersed TGN staining (bottom panel), there was still strong co-localization between the TGN marker and tetherin.

### Vpu and HIV-2 Env alter the trafficking of tetherin between the cell surface and the TGN

Tetherin is recycled between the plasma membrane and the TGN by AP-2 mediated endocytosis, followed by AP-1 mediated retrotransport to the TGN [21,30]. Since the number of cells exhibiting an intracellular tetherin concentration significantly increased in the presence of Vpu or ROD10 Env, we speculated that this could reflect either an increase in the rate of tetherin endocytosis from the surface and retrotransport to the TGN or, alternatively, be caused by a block in tetherin transport from the TGN to the cell surface.

**Figure 8:**
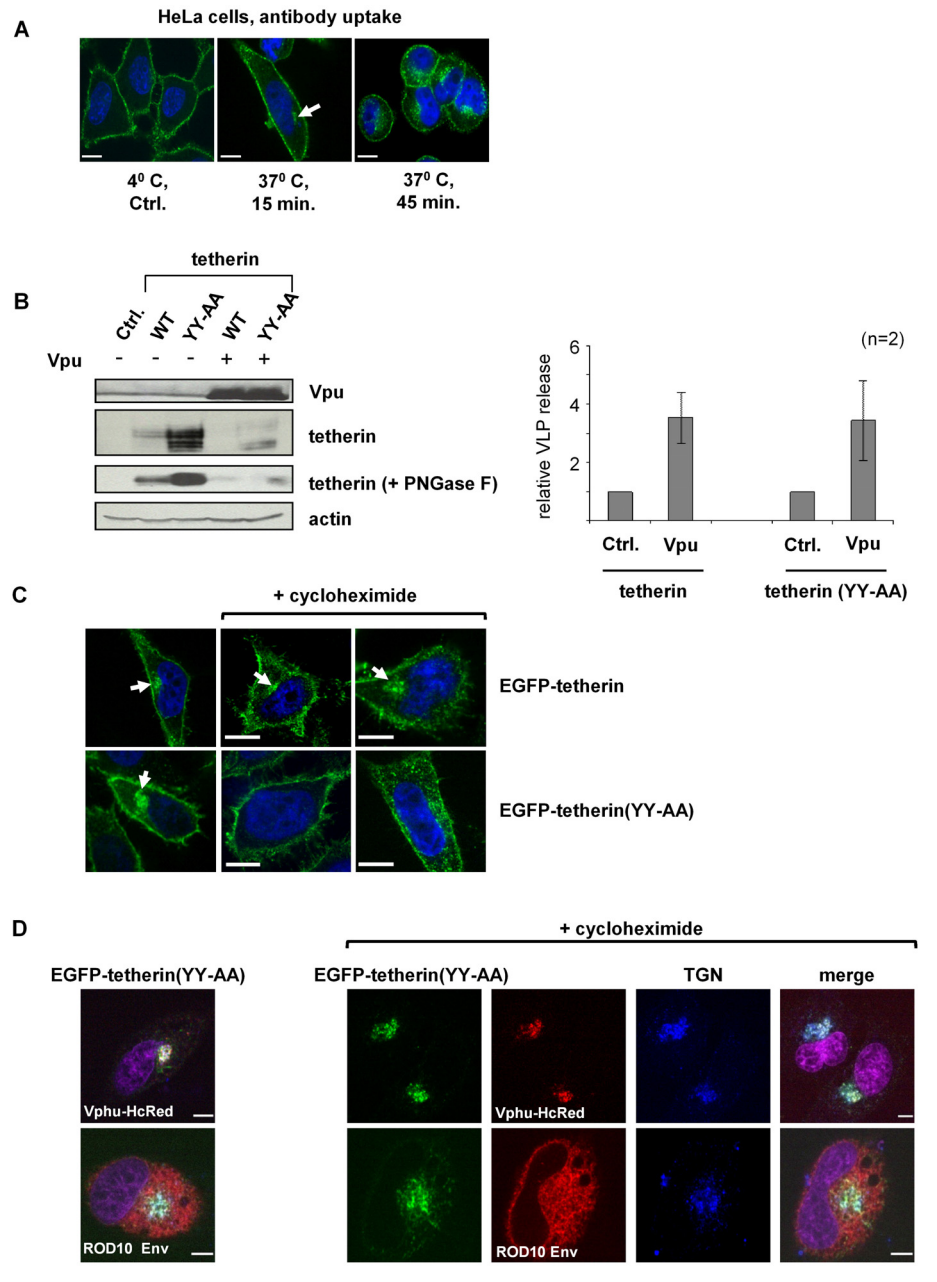
**Analysis of perinuclear concentration of tetherin by Vpu and ROD10 Env**. **(A) **HeLa cells were incubated with anti-tetherin antibody at 4°C for 1 hour, followed by incubation at 37°C for either 15 or 45 minutes to allow endocytosis of antibody-tetherin complexes. The antibody was found in a perinuclear region by 15 minutes (arrowed), but became more diffuse by 45 minutes, suggesting that tetherin quickly exits this compartment and recycles back to the plasma membrane. Scale bars represent 10 μM. **(B) **HIV-1 VLPs produced in 293A cells in the presence of 100 ng of wild-type (WT) or YY-AA tetherin, with and without co-transfection of 2 μg Vpu (pCMV-Vphu). Expression of proteins was confirmed by Western blotting with specific antibodies; tetherin levels were visualized for both untreated and PNGase F treated cell lysates. Mean +/- SEM fold-enhancement of VLP release by Vpu is shown in presence of tetherin or tetherin (YY-AA), n = 2. **(C) **HeLa cells expressing EGFP-tetherin or EGFP-tetherin(YY-AA) were incubated for 1.5 hours, with or without 20 μg/ml cycloheximide, and subsequently fixed, permeabilized, and stained with anti-EGFP antibody. In the absence of drug, both WT and YY-AA EGFP-tetherin proteins were found at the cell surface and in a perinuclear region (arrowed). After cycloheximide treatment, only EGFP-tetherin produced this population, with EGFP-tetherin(YY-AA) forming a more punctuate pattern, dispersed throughout the cytoplasm. Two different cells are shown for the drug treatment. Scale bars represent 10 μM. **(D) **HeLa cells were co-transfected with 60 ng EGFP-tetherin(YY-AA) and 2 μg of expression plasmids for either Vphu-HcRed or ROD10 Env. Cells were incubated with or without cycloheximide, as above, and subsequently fixed, permeabilized, and stained with anti-EGFP (green), anti-TGN46 (blue) and anti-HIV-2 Env (red) antibodies. Vpu was detected by HcRed expression (red). EGFP-tetherin(YY-AA) was concentrated in a perinuclear compartment by Vpu or ROD10 Env, overlapping with the TGN marker, irrespective of cycloheximide treatment. Scale bars represent 10 μM.

To confirm that human tetherin recycles between the plasma membrane and an intracellular pool, we labeled cell-surface tetherin with antibody and determined its cellular localization after 15 and 45 minutes incubation at 37°C (Figure 8A). Under these conditions, endocytosed antibody-labeled tetherin was clearly visible in a compact perinuclear region in about 10% of the cells after 15 minutes incubation. By 45 minutes, intracellular staining was observed in all cells, although in a larger and more diffuse pool, which is consistent with tetherin being recycled back to the cell surface. As a control, cells incubated at 4°C displayed no internalized protein-antibody complexes. In cells also expressing Vpu or ROD10 Env, we were not able to detect any endocytosed tetherin-antibody complexes using this assay (data not shown), which is likely a consequence of the fact that both proteins decrease the steady-state levels of cell surface tetherin, so that insufficient antibody was bound to be detected in the assay.

We next asked whether the natural pathway of tetherin endocytosis was necessary for the observed perinuclear redistribution of tetherin in the presence of Vpu or ROD10 Env. We generated a mutant of tetherin with alanine substitutions of a double tyrosine motif in the N-terminal cytoplasmic tail of the protein (YY-AA) that has previously been reported to interact with AP-1 and AP-2, and whose mutation stabilizes tetherin at the cell surface [21,26,30]. Mutant (YY-AA) was examined for its ability to restrict HIV-1 VLP release from 293A cells, where it was found to be fully functional, and even slightly more restrictive than the wild-type (data not shown). Western blotting revealed that mutant (YY-AA) was present at higher levels in cell lysates, suggesting stabilization of the protein (Figure 8B). Both Vpu and ROD10 Env were able to effectively counteract the YY-AA mutant (Figure 8B and data not shown). In addition, Vpu maintained the ability to promote the degradation of both the WT and YY-AA proteins (Figure 8B). These observations are in agreement with a recently published study showing Vpu counteracts the YY-AA mutant efficiently [26]. We conclude that the natural endocytosis pathway used by tetherin is not required for either virus release restriction or its ablation by Vpu or HIV-2 Env.

To facilitate visualization, we constructed an EGFP-tagged version of the YY-AA tetherin mutant. Under conditions where population of the TGN with newly synthesized proteins was blocked (cycloheximide treatment), this mutant failed to concentrate in a perinuclear region (Figure 8C). This suggests that the YY-AA mutant is unable to recycle back to a perinuclear pool from the cell surface by the normal AP-2 and AP-1-dependent pathways. Instead, the YY-AA mutant was observed to be dispersed in vesicles throughout the cytoplasm, presumably caused by internalization using other pathways. In contrast, the wildtype EGFP-tetherin was still able to form a perinuclear concentration, irrespective of the presence of cycloheximide (Figure 8C).

Next, we examined the consequences of co-expression of either Vpu or the ROD10 Env on the cellular distribution of the EGFP-tetherin (YY-AA) mutant. Independently of the presence of cycloheximide, we observed a complete loss of the cell surface protein and strong perinuclear accumulation which overlapped with a marker for the TGN (Figure 8D). Taken together, these findings are consistent with a model where cell surface tetherin is depleted in the presence of Vpu or ROD10 Env, and the protein is sequestered intracellularly in a perinuclear compartment that includes the TGN. Tetherin in this compartment could represent either newly synthesized tetherin that is trapped in the TGN *en route *to the plasma membrane, and/or protein that has been internalized from the plasma membrane by a pathway that does not use the natural tetherin endocytosis mechanism and is dependent on expression of these viral anti-tetherin factors.

## Discussion

BST-2/tetherin inhibits the release of enveloped viruses from the surface of infected cells and appears to be an intrinsic cellular anti-viral defense [31]. Although tetherin's activity was initially identified against Vpu-defective HIV-1 particles, it has now been shown to restrict a broad range of enveloped viruses [10,12] and the growing list of viral tetherin antagonists so far identified includes HIV-1 Vpu [3,4], HIV-2 Env [13], SIV Nef [14-17], KSHV K5 [19] and Ebola GP [12]. These observations suggest that tetherin exerts a significant antiviral effect against enveloped viruses that successful pathogens must overcome.

The unusual topology of tetherin, existing as a dimer with two different membrane anchoring domains per monomer [20], has led to the suggestion that it could simultaneously be anchored in both host and viral membranes and thereby physically tether virions to the plasma membrane [3]. This suggests that simply removing tetherin from the cell surface could be the basis for the action of some, or all, anti-tetherin factors [4]. Several viral proteins are already known that block aspects of the host immune response by targeting cell surface proteins. For example in HIV-1, Vpu simultaneously binds to CD4 and βTrCP in the ER to mediate ubiquitinylation and proteasomal degradation of CD4 [32], while Nef relocalizes MHC-I to the TGN and/or reroutes newly synthesized MHC-I to lysosomes by physically connecting MHC-I to AP-1 [33,34]. In KSHV, the K3 and K5 proteins are E3 ubiquitin ligases that enhance internalization of several cell surface proteins and target them for endo-lysosomal degradation [35].

Analysis of tetherin's cellular distribution in HeLa cells by FACS and confocal microscopy identified a portion of the protein at the plasma membrane, with an additional concentration in a perinuclear region that co-stained with markers for the TGN and late endosomes. The cell surface fraction was significantly depleted by the expression of the Vpu and the ROD10 Env, but not by mutants of the HIV-2 Env that were unable to counteract tetherin restriction or enhance the release of HIV-1 particles. In addition, we observed that both Vpu and ROD10 Env caused a significant redistribution of intracellular tetherin into the TGN, and that Vpu alone also increased the association of tetherin with recycling endosomes.

Vpu, but not ROD10 Env, also reduced total steady-state levels of tetherin. Since Vpu is known to recruit βTrCP to target CD4 for proteasomal degradation, it has been suggested that Vpu also uses this interaction to degrade tetherin, and Vpu degradation of HA-tagged tetherin expressed in 293T cells has previously been reported to be sensitive to proteasomal inhibitors [24,36]. However, other studies have shown that proteasomal degradation is not required for Vpu's ability to enhance virus release [23,37], and that the proteasomal inhibitor MG132 has only a modest effect on the ability of Vpu to remove tetherin from the cell surface [4], or to reduce total cellular tetherin levels [25]. As an alternative mechanism, it is possible that Vpu uses the interaction with βTrCP to target tetherin to an endo-lysosomal pathway. In support of this model, Mitchell *et al. *[27] reported that Vpu removal of tetherin from the cell surface was sensitive to βTrCP downregulation or dominant-negative interference, could be rescued by bafilomycin treatment and required the conserved di-serine motif in Vpu's tail that is known to interact with βTrCP. Similarly, Douglas *et al. *reported a 50% decrease in Vpu's anti-tetherin activity following βTrCP depletion or mutation of the two serines, and that the degradation of cellular tetherin by Vpu was blocked by concanamycin A [25]. The partial effects observed in both of these studies suggest that these βTrCP-mediated effects may not be the only mechanism used by Vpu to counteract tetherin. In support of this hypothesis, other reports have described little or no requirement for the two serine residues in Vpu's tail in order to stimulate HIV-1 release [23,38,39].

An alternative, or additional, mechanism suggested by our observations is that Vpu counteracts tetherin by sequestering it in an intracellular compartment that overlaps with markers for the TGN. Such an accumulation could involve trapping of newly synthesized tetherin in the TGN, as well as protein that has been recycled from the cell surface. The fact that we observed this accumulation even for a tetherin mutant (YY-AA) that is defective in recycling suggests that Vpu can indeed sequester the newly synthesized, non-recycled tetherin. Additionally, we observed strong co-localization of Vpu and tetherin in the TGN, and others have demonstrated an interaction between the two proteins by co-immunoprecipitation [25,26,40]. Retention of Vpu in the TGN has been reported to increase its ability to enhance virus release, while a Vpu mutant that mislocalized outside the TGN had reduced activity [29]. A TGN trapping mechanism to counteract host anti-viral defenses has precedent in the HIV-1 Nef protein, which disrupts trafficking of MHC-I from the TGN to the plasma membrane [33,34].

We propose a model whereby Vpu both retains tetherin in the TGN and simultaneously marks it for degradation by recruiting β-TrCP to mediate its ubiquitinylation. In a similar manner, Vpu uses β-TrCP to affect the proteasomal degradation of CD4 that has been trapped in the ER by its interaction with gp120 [41]. The apparent discrepancies in recent reports of how Vpu might target tetherin [23-27,38,42] are consistent with the possibility of more than one mechanism being used to ensure the removal of tetherin from the site of HIV-1 budding. Furthermore, the predominant effect observed may differ between cell types, and under different expression conditions of either endogenous or exogenously expressed tetherin.

We also examined the anti-tetherin activity of the HIV-2 ROD10 Env protein. This protein had no significant effect on total cellular tetherin levels by Western analysis. Despite this, the ROD10 Env proved to be a potent inhibitor of tetherin restriction that also sequestered tetherin in the TGN, although, unlike Vpu, the majority of the Env protein was not co-localized with tetherin at this site. In agreement with our observations, recent studies have also shown that both HIV-2 Env and SIVtan Env sequester tetherin in the TGN [13,43].

In previous work we have shown that the ability of the ROD10 Env to enhance virus release requires a membrane-proximal tyrosine motif (Y707) in the cytoplasmic tail of the Env that promotes its endocytosis and interacts with AP-2 [44]. These findings appeared to reflect a requirement for Env trafficking signals, since the dependence on AP-2 could be removed by substituting the cytoplasmic tail of ROD10 Env with the same region from the MLV Env protein [44]. Here, we have further shown that mutation of Y707, or expression of a defective Env from the ROD14 strain, also blocked the removal of tetherin from the cell surface. Previous studies have mapped the defect in the ROD14 Env to a single amino acid change at position 598 in the ectodomain of its TM protein [7,45]. In other work, we have found that a tetherin derivative containing just the ectodomain of the protein linked to the transmembrane and cytoplasmic domains of the transferrin receptor is still able to inhibit virus release, and in a manner that can be counteracted by the HIV-2 Env, but not by Vpu [46]. This suggests that a physical interaction could be occurring between the ectodomains of the Env and tetherin, and that such a complex could subsequently be removed from the cell surface and directed towards a perinuclear compartment using HIV-2 Env-mediated endocytosis. Diverting tetherin from its normal recycling pathway in this manner could eventually deplete cell surface levels.

## Conclusions

Previous studies have suggested that Vpu counteracts tetherin by recruitment of β-TrCP, leading to either proteasomal or endo-lysosomal degradation [24-27,42]. Our findings suggest an additional mechanism whereby Vpu and the ROD10 Env can also remove tetherin from the cell surface by redirecting the protein to a perinuclear compartment. This redirection was independent of tetherin's normal trafficking pathway, suggesting that the mechanism could instead involve direct protein-protein interactions with the viral anti-tetherin factors and utilization of the trafficking machinery recruited by these two different HIV proteins. Redundant mechanisms to counteract tetherin have evolved within the primate lentiviruses, with at least three known anti-tetherin factors so far identified in the Vpu, Env and Nef proteins. Our data suggest that Vpu itself could be using more than one mechanism to block tetherin's activity.

## Materials and methods

### Cell lines

HeLa cells were obtained from the American Type Culture Collection, 293A cells were obtained from Qbiogene/MP Biomedicals (Irvine, CA), and HeLa-CD4 P4.R5 cells were obtained from Ned Landau (NYU School of Medicine). All cell lines were maintained in D10 media: Dulbecco's modified Eagle's medium (DMEM) (Mediatech, Herndon, VA) supplemented with 10% fetal bovine serum (FBS) (Mediatech) and 2 mM glutamine (Gemini Bio-Products, West Sacramento, CA) (D10 media).

### Plasmids

Plasmid pHIV-1-pack expresses the HIV-1 Gag-Pol and Rev [7]. Plasmid pcDNA-Vphu encodes a human codon-optimized form of NL4-3 Vpu (Vphu) [47], kindly provided by Klaus Strebel (NIH). VphuHcRed expresses Vphu with a C-terminus fusion of HcRed [48], and was obtained from Paul Spearman (Emory University). HIV-2 Env expression plasmids from isolates ROD10 and ROD14, and the ROD10(Y_707_A) mutant, have been previously described [7,44]. The HIV-1_NL4-3 _proviral clone was obtained from the AIDS Research and Reference Reagent Program (ARRRP) and the HIV-2_ROD10 _proviral clone was a kind gift from Klaus Strebel (NIH). A BST-2/tetherin expression plasmid (pCMV6-XL5-Bst2) was obtained from Origene (Rockville, MD) and an N-terminal EGFP-tagged version, (pEGFP-tetherin), was made by cloning into vector pEGFP-C1 (Clontech, Mountain View CA), with the addition of the 12 amino acid linker, GHGTGSTGSGSS, between the two proteins. A mutant version with tyrosine to alanine substitutions at positions 6 and 8, EGFP-tetherin(YY-AA), was created by site-directed mutagenesis.

### Production and analysis of HIV-1 VLPs

HIV-1 VLPs were generated in HeLa or 293A cells by transient transfection of 80-90% confluent cultures with 10 μg of plasmid pHIV-1-pack (expresses Gag-Pol and Rev), together with 2 μg of Vpu or HIV-2 Env expression plasmids, using Lipofectamine 2000 (Invitrogen, Carlsbad, CA), essentially as previously described [7,16]. Cell lysates were harvested and viral particles were collected from the supernatant after 24 h and analyzed by Western blotting, as previously described [44]. HIV-1 p24-reacting proteins were detected using rabbit HIV-1_SF2 _p24 antiserum (ARRRP) at a 1:3,000 dilution, and expression of co-transfected proteins was detected with specific antisera; 1:1,000 dilution of rabbit HIV-1_NL4-3 _Vpu antiserum (ARRRP, deposited by Frank Maldarelli and Klaus Strebel), 1:1000 dilution of rabbit HIV-2_ST_-gp120 antiserum (ARRRP, deposited by Raymond Sweet), 1:3000 dilution of rabbit anti-GFP (Invitrogen, Carlsbad, CA). The secondary antibody used was horseradish peroxidase (HRP)-conjugated goat anti-rabbit IgG (1:10,000) (Pierce, Rockford, IL). Specific proteins were visualized using the enhanced chemiluminescence (ECL) detection system (Amersham International, Arlington Heights, IL). Exposed and developed films were scanned and quantified using the public domain NIH ImageJ software. The intensities of p24-reacting bands on Western blots were measured, and the ratio of the signal in virions:lysates was obtained. The fold-enhancement of virus budding was calculated by normalizing all values to the pHIV-1-pack only control.

### Tetherin immunoblotting

Tetherin was detected by Western blotting of cell lysates, normalized to 15 μg protein per sample. Samples were also deglycosylated by incubation for 5 min in Denaturing Buffer (NEB, Ipswich, MA) at 90°C followed by incubation at 37°C for 3 hrs with 500 Units PNGaseF (NEB) in PNGaseF Buffer supplemented with 1% NP-40 (NEB). Tetherin was detected using a 1:20,000 dilution of polyclonal rabbit anti-BST-2 (ARRRP, deposited by Klaus Strebel), followed by a 1:10,000 dilution of HRP-conjugated goat anti-rabbit IgG (Pierce, Rockford, IL). Specific bands were visualized by ECL.

### Confocal microscopy

HeLa or 293A cells were transfected with specific expression plasmids in 10 cm dishes using Lipofectamine 2000 (Invitrogen). The amounts of each plasmid transfected were 100-300 ng of EGFP-tetherin, 2 μg of each of the Vpu or HIV-2 Env expression plasmids, or 8 μg of proviral clone plasmids. Eighteen-24 hrs later, cells were seeded on coverslips coated with poly-L-lysine (Sigma-Aldrich, St. Louis, MO). The cells were incubated for an additional 24 hrs at 37°C and processed for antibody staining. For analysis of surface expression, cells were placed at 4°C for 20 mins, incubated with fresh D10 plus antibody at 4°C for 30 minutes, washed with PBS, fixed with 4% paraformaldehyde for 20 minutes at room temperature, and washed three times in PBS. To visualize intracellular proteins, cells were subsequently permeabilized for 10 mins in 0.1% Triton X-100 at room temperature, and washed three times in PBS. Mouse anti-GFP monoclonal antibody (Invitrogen) was used at a 1:500 dilution. Tetherin was detected using a polyclonal mouse anti-BST-2 antibody, MaxPab H00000684-B02P (Abnova) at a 1:150 dilution. HIV-2 Env proteins were detected using a 1:1,000 dilution of rabbit polyclonal serum against the HIV-2_ST _SU protein (ARRRP). Vpu was detected using rabbit HIV-1_NL4-3 _Vpu antiserum (ARRRP) at 1:1,000 dilution. The trans-Golgi network was detected using a sheep polyclonal anti-TGN46 antibody (Serotec, Oxford, UK) at 1:1000 dilution. The human transferrin receptor 1 (hTfR1) was detected with a monoclonal mouse-anti-CD71 antibody (Santa Cruz Biotechnology, Santa Cruz, CA) at 1:60 dilution. The conjugated secondary antibodies used were donkey anti-mouse AlexaFluor 488, donkey anti-rabbit AlexaFluor 594, donkey anti-sheep AlexaFluor 594, donkey anti-goat AlexaFluor 647 and donkey anti-sheep AlexaFluor 647 (Invitrogen). Staining of endocytosed transferrin was performed by starving cells in serum free DMEM for 1 hr at 37°C, followed by the addition of transferrin from human serum conjugated with AlexaFluor 647 (Invitrogen) at 50 μg/ml in D10 for 30 minutes at 37°C, followed by fixation and permeabilization as described above. Processed cells were mounted in Prolong Gold antifade reagent with DAPI (Invitrogen). Images were acquired with the PerkinElmer Ultraview ERS laser spinning disk confocal imaging system at 100× magnification (PerkinElmer, Waltham, MA) and processed using Volocity software (Improvision, PerkinElmer) and Adobe Photoshop Creative Suite 2.

### Co-localization analyses

Confocal images were analyzed using the co-localization plugin of the public domain NIH ImageJ software. This uses intensity correlation analysis, where the distribution of the intensity value for each pixel in a channel is plotted against the product of the difference of the mean (PDM) of the two channels. The output is shown as a pseudocolor graph, with areas of co-localization having a positive PDM (orange). In addition, Pearson correlation coefficients of signal co-localization were calculated using the JACoP plug-in of ImageJ. A value of +1 reflects perfect correlation and -1 is complete separation of the proteins. When Pearson coefficients were calculated for intracellular staining, the cell surface fraction of the protein was masked before analysis.

### Flow cytometry

Cell surface tetherin was detected by incubation of cells with the HM1.24 murine monoclonal antibody (Chugai Pharmaceutical Co., Kanagawa, Japan) followed by goat anti-mouse IgG conjugated to allophycocyanin, as previously described [4].

### Inhibition of protein expression

Transfected cells were seeded onto cover slips and, 24 hrs later, incubated for 1.5 hours in D10 plus 200 μg/ml cyclohexamide (Sigma-Aldrich). Cells were washed in phosphate-buffered saline (PBS) and processed for confocal microscopy as described above.

## Competing interests

The authors declare that they have no competing interests.

## Authors' contributions

HH and LAL carried out most of the experimental work, participated in the analysis of results and contributed to writing the manuscript, SJY, JEO and JCG performed experimental work and interpreted data, CME contributed to discussion and writing the manuscript, PMC conceived the study, participated in its design and co-ordination and helped to write the manuscript. All authors read and approved the final manuscript.
