# HIV-1 Vpu and HIV-2 Env counteract BST-2/tetherin by sequestration in a perinuclear compartment

**DOI:** 10.1186/1742-4690-8-85

**Published:** 2011-10-25

**Authors:** Heiko Hauser, Lisa A Lopez, Su Jung Yang, Jill E Oldenburg, Colin M Exline, John C Guatelli, Paula M Cannon

**Affiliations:** 1 Keck School of Medicine of the University of Southern California, California Los Angeles, CA 90089, USA; 2 University of California San Diego, 9500 Gilman Dr., La Jolla, CA 92093, California, USA

## Correction

A confocal image in Figure 3B of Hauser et al. 2010 [1], showing TGN staining of Vphu-HcRed expressing cells (middle row,) was incorrect. This image has now been replaced with the correct image.

## Supplementary Material

Additional file 1 **Updated versions of Figure 3 of Hauser et al. 2010 **[1]. Redistribution of tetherin to an intracellular compartment by HIV anti-tetherin factors. **(A) **The percentage of HeLa cells displaying tetherin concentrated in a perinuclear compartment (PNC) was calculated for 100 cells, from either control (Ctrl.) cells or cells transfected with 2 μg of Vpu or ROD10 Env expression plasmids. Mean +/- SEM is shown for n = 2 independent experiments. **(B) **HeLa cells transfected with either Vpu (Vphu-HcRed) or ROD10 Env, showed increased concentration of tetherin in a perinuclear compartment (arrowed), that co-stained with the TGN marker, TGN46. The triple color merged image is shown. Scale bars represent 10 μM. Click here for file

## References

[B1] Hauser Heiko Lopez Lisa A Yang Su J Oldenburg Jill E Exline Colin M Guatelli John C Cannon Paula M HIV-1 Vpu and HIV-2 Env counteract BST-2/tetherin by sequestration in a perinuclear compartment Retrovirology 2010 7 51 10.1186/1742-4690-7-51 20529266 PMC2890665

