# Supplementary figures and images for: HIV-1 Vpu and HIV-2 Env counteract BST-2/tetherin by sequestration in a perinuclear compartment
Source: Retrovirology. 2011 Oct 25;8:85. doi: 10.1186/1742-4690-8-85 (PMC3214836; doi:10.1186/1742-4690-8-85)

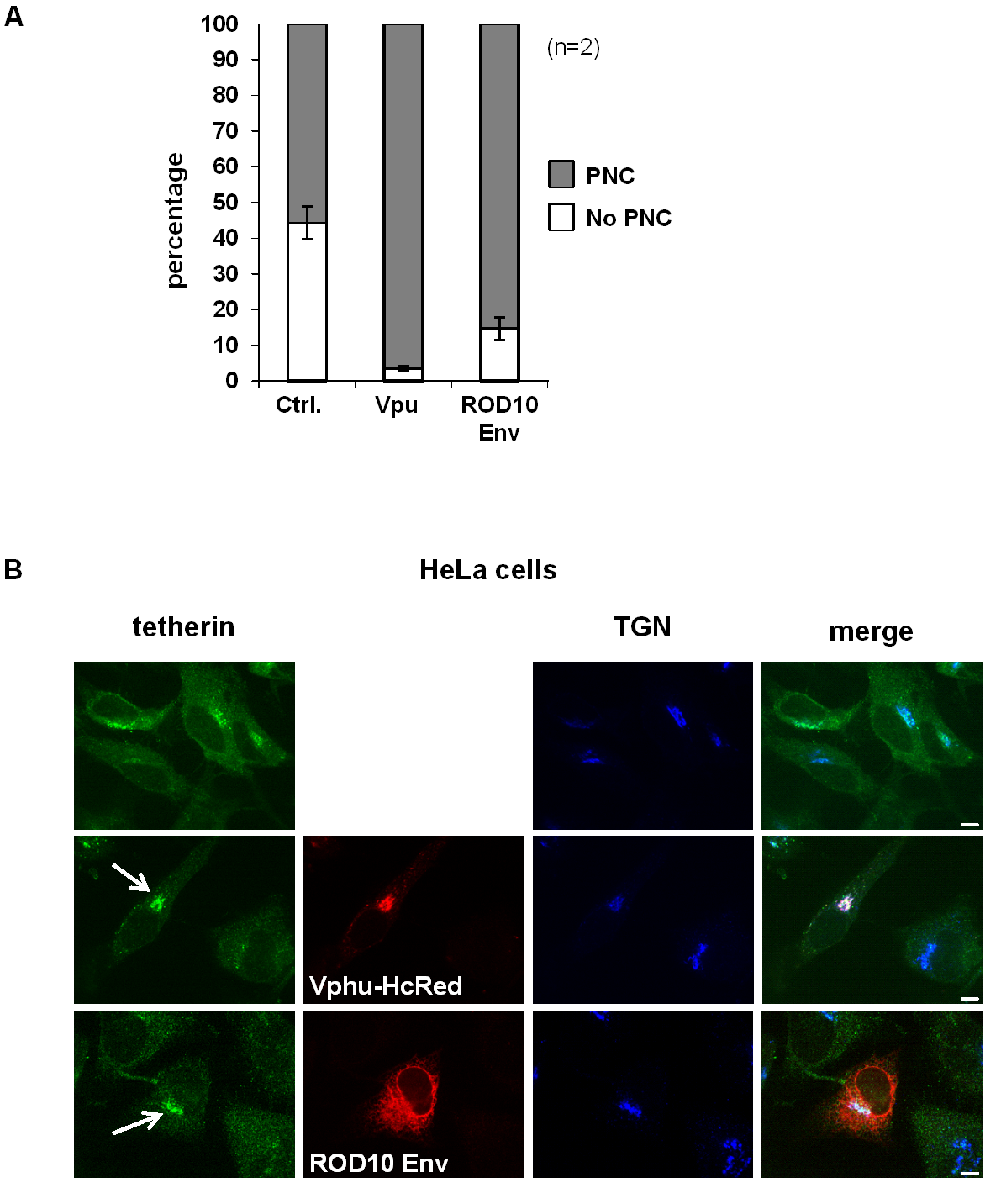

Supplement: Additional file 1 — Updated versions of Figure 3 of Hauser et al. 2010 [1]. Redistribution of tetherin to an intracellular compartment by HIV anti-tetherin factors. (A) The percentage of HeLa cells displaying tetherin concentrated in a perinuclear compartment (PNC) was calculated for 100 cells, from either control (Ctrl.) cells or cells transfected with 2 μg of Vpu or ROD10 Env expression plasmids. Mean +/- SEM is shown for n = 2 independent experiments. (B) HeLa cells transfected with either Vpu (Vphu-HcRed) or ROD10 Env, showed increased concentration of tetherin in a perinuclear compartment (arrowed), that co-stained with the TGN marker, TGN46. The triple color merged image is shown. Scale bars represent 10 μM. [file 1742-4690-8-85-S1.TIFF]
